# Genetic Diagnosis of Cockayne Syndrome

**DOI:** 10.15844/pedneurbriefs-34-9

**Published:** 2020-12-02

**Authors:** Rifali Patel

**Affiliations:** 1 Department of Pediatric Neurology, MercyOne Children's Hospital, Des Moines, IA

**Keywords:** Cockayne Syndrome, DNA repair deficiency, Activating Transcription Factor 3

## Abstract

Researchers from the University of Strasbourg investigated whether defective transcription of ATF3 responsive genes is a marker for Cockayne Syndrome (CS).

Researchers from the University of Strasbourg investigated whether defective transcription of ATF3 responsive genes is a marker for Cockayne Syndrome (CS). CS is a rare genetic disorder caused by pathogenic variants (dysfunction) in the *CSA* and *CSB* genes. CS patients exhibit mild photosensitivity and severe neurological problems. *ATF3* is over-expressed following cellular stress and closely linked to motor and sensory neuron degradation and sometimes used as a neuronal damage marker. When activated during stress, *ATF3* will repress up to 5000 genes for a short period. In CS cells, *CSA* and *CSB* dysfunction impairs the degradation of the chromatin-bound *ATF3*, leading to a permanent transcriptional arrest as observed by immunofluorescence and ChIP followed by RT-PCR. Currently, CS diagnosis is based on CS cells' inefficiency to recover RNA synthesis upon genotoxic (e.g., UV) stress. This study has demonstrated results of ChIP-seq of Pol II and *ATF3* promoter occupation analysis and RNA sequencing-based gene expression profiling in CS cells, immunofluorescence study of ATF3 protein stability, and quantitative RT-PCR screening in 64 patient cell lines. The results confirm that the analysis of a few *ATF3* dependent genes, for example, *CDK5RAP2*, *NIPBL,* and *NRG1*, could serve as prominent molecular markers to discriminate between CS and non-CS patient's cells. Utilizing this assay can significantly simplify the CS diagnostic procedure's timing and complexity compared to the currently available methods. [1]

COMMENTARY. The phenotype and diagnosis of CS are complex. Clinical features of CS include cachectic dwarfism, severe neurological manifestations including microcephaly, cognitive deficits, pigmentary retinopathy, cataracts, sensorineural deafness, which overlaps with progeria and xeroderma pigmentosum (XP) with average life expectancy up to the second decade [2]. CS is inherited as an autosomal recessive genetic trait. The genes are responsible for CS -type I mapped to chromosome 5, known as ERCC8, and for CS-type II on chromosomal locus 10q11, known as ERCC6. Mutations in ERCC6 account for about 75% of cases, while mutations in ERCC8 cause about 25% of cases [2]. So far, the molecular bases of a defect in transcription and related coupled nucleotide excision repair have been evaluated for diagnostic purposes, in addition to clinical features. The traditional prenatal diagnosis of CS is performed with the reduced recovery of DNA‐synthesis in UV‐irradiated cultured chorionic villus cells or amniocytes, which is time-consuming [3]. The new ATF3 Promote analysis technique includes a multistep approach with treating cell lines with UVC treatment followed by immunostaining of DNA testing with ChIP seq, RNA seq, and NG Sequencing of CS genes. ATFE3 Promote assay is sensitive to test most involved genes in CS, including CSA, CSB, XPB, XPD, XPG, in a time-sensitive manner, especially in prenatal diagnosis or in early stages of disease in the absence of any identified molecular defect. In response to cellular stress, ATF3 immediate early gene activated and rapidly transiently targets genes harboring a CRE/ATF binding site to repress their expression. This test detects mutations in CDK5RAP2, NRG1, and NIPBL genes involved in neurodevelopmental or neurodegenerative processes and confirms the same pattern in all proven CS / XP patients neurological symptoms or but not observed in mutations in XP genes associated with dermatological symptoms. As of now, the limitation of the test is a small number of patients. For the neurologist community, using ATF3 Promote analysis can improve diagnostic yield when conventional testing with the reduced recovery of DNA synthesis analysis is nonconclusive, especially in the early stages of CS and highly suspected CS cases with neurological and non-neurological phenotypes [1].

## Disclosures

The author has declared that no competing interests exist.
